# 

**Published:** 1874-06

**Authors:** 


### DENTAL REGISTER,

A Monthly Journal Devoted to
the Interests of the Profession.

EDI ED BY J. TAFT, D. D. S.

AND

PUBLISHED BY SPENCER, CROCKER & CO.,
OHIO DENTAL DEPOT,
168 Race Street, Cincinnati, Ohio.

TERMS: $2.50 Per Annum, in Advance; Single Copies 25c.

